# Supplementary material for: CK2-induced cooperation of HHEX with the YAP-TEAD4 complex promotes colorectal tumorigenesis
Source: Nat Commun. 2022 Aug 25;13:4995. doi: 10.1038/s41467-022-32674-6 (PMC9411202; doi:10.1038/s41467-022-32674-6)
Supplement: Supplementary file 1 — Supplementary Information [file 41467_2022_32674_MOESM1_ESM.pdf]

Supplementary Information

for

CK2-induced Cooperation of HHEX with the YAP-TEAD4 Complex Promotes Colorectal Tumorigenesis

Yuegui Guo[1,4,5], Zhehui Zhu[1,4,5], Zhenyu Huang[1,4], Long Cui[1,4], Wei Yu[2], Wanjin Hong[3], Zhaocai Zhou[2]*, Peng Du[1,4]*, Chen-Ying Liu[1,4]*

1 Department of Colorectal and Anal Surgery, Xinhua Hospital, Shanghai Jiao Tong University School of Medicine, Shanghai 200092, China

2 State Key Laboratory of Genetic Engineering, School of Life Sciences, Zhongshan Hospital, Fudan University, Shanghai 200438, China

3 Institute of Molecular and Cell Biology, Agency for Science, Technology and Research (A*STAR), 61, Biopolis Drive, Proteos, Singapore 138673, Singapore

4 Shanghai Colorectal Cancer Research Center, Shanghai 200092, China

5 These authors contribute equally.

*Correspondence and requests for materials should be addressed to Zhaocai Zhou (email: zhouzhaocai@fudan.edu.cn) or Peng Du (email: dupeng@xinhuamed.com.cn) or Chen-Ying Liu (email: liuchenying@xinhuamed.com.cn).

This supplementary file contains Supplementary Figure 1 to 9 and Supplementary Table 1 to 2.

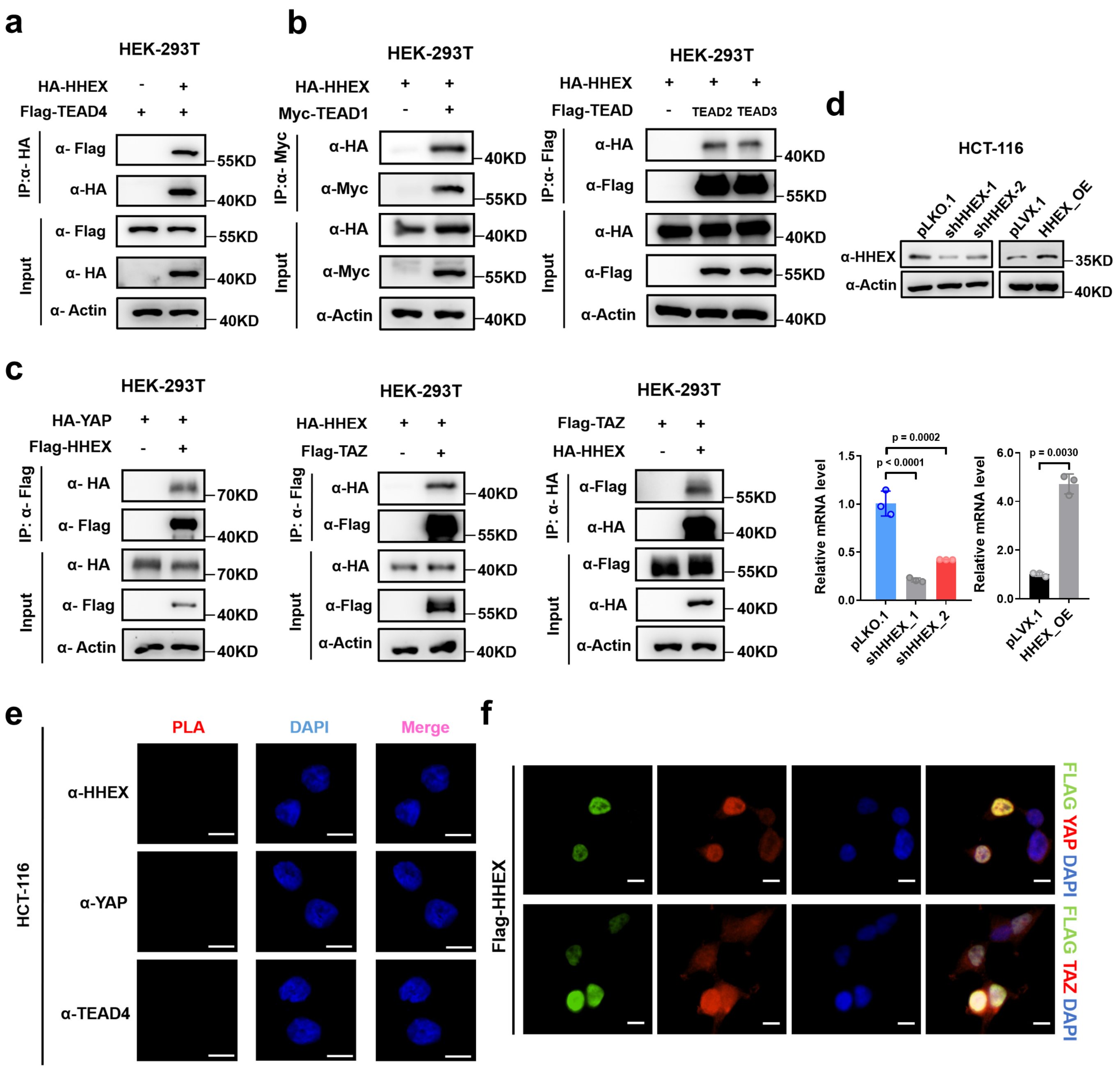

Supplementary Fig. 1. HHEX interacts with TEADs, YAP and TAZ

a Co-IP of exogenous HA-HHEX and FLAG-TEAD4 in HEK-293T cells. **b** Co-IP of exogenous HA-HHEX and Myc-TEAD1 in HEK-293T cells (left). Co-IP of exogenous HA-HHEX and FLAG-TEAD2 or FLAG-TEAD3 in HEK-293T cells (right).**c** Co-IP of exogenous FLAG-HHEX and HA-YAP in HEK-293T cells, and reciprocal co-IP of exogenous HA-HHEX and FLAG-TAZ in HEK-293T cells. **d** Generation of HCT-116 cells with stable *HHEX* knockdown or overexpression. Western blot and qPCR analysis were performed to determine the *HHEX* mRNA level. Data are mean ± SD, n=3 biologically independent samples per group. One-way ANOVA and Two-tailed Welch's t-test were performed to assess statistical significance for the experiments with 3 groups and 2 groups, respectively. **e** The specificity of the PLA signals (red) was assessed by using single anti-YAP, anti-TEAD4 or anti-HHEX antibody in the PLA assay in the HCT-116 cells. Scale bars, 10 μm. **f** Immunofluorescence analysis of colocalization of HHEX (green) with YAP/TAZ (red) in the nucleus in HCT-116 cells. Nuclei were stained with DAPI (blue). HCT-116 cells were transfected with FLAG-HHEX for 36 hr before processing for immunofluorescence staining using anti-FLAG and anti-YAP/TAZ antibodies. Scale bar, 50 μm. In these co-IP assays, HEK-293T cells were cotransfected with the indicated plasmids. – indicates no transfection; + indicates transfection. Co-IP was performed with anti-FLAG M2, anti-HA or anti-Myc magnetic beads. Immunoprecipitates were analyzed by western blotting using the indicated antibodies. These data (**a-c**, **e-f**) are representative of 3 independent experiments.

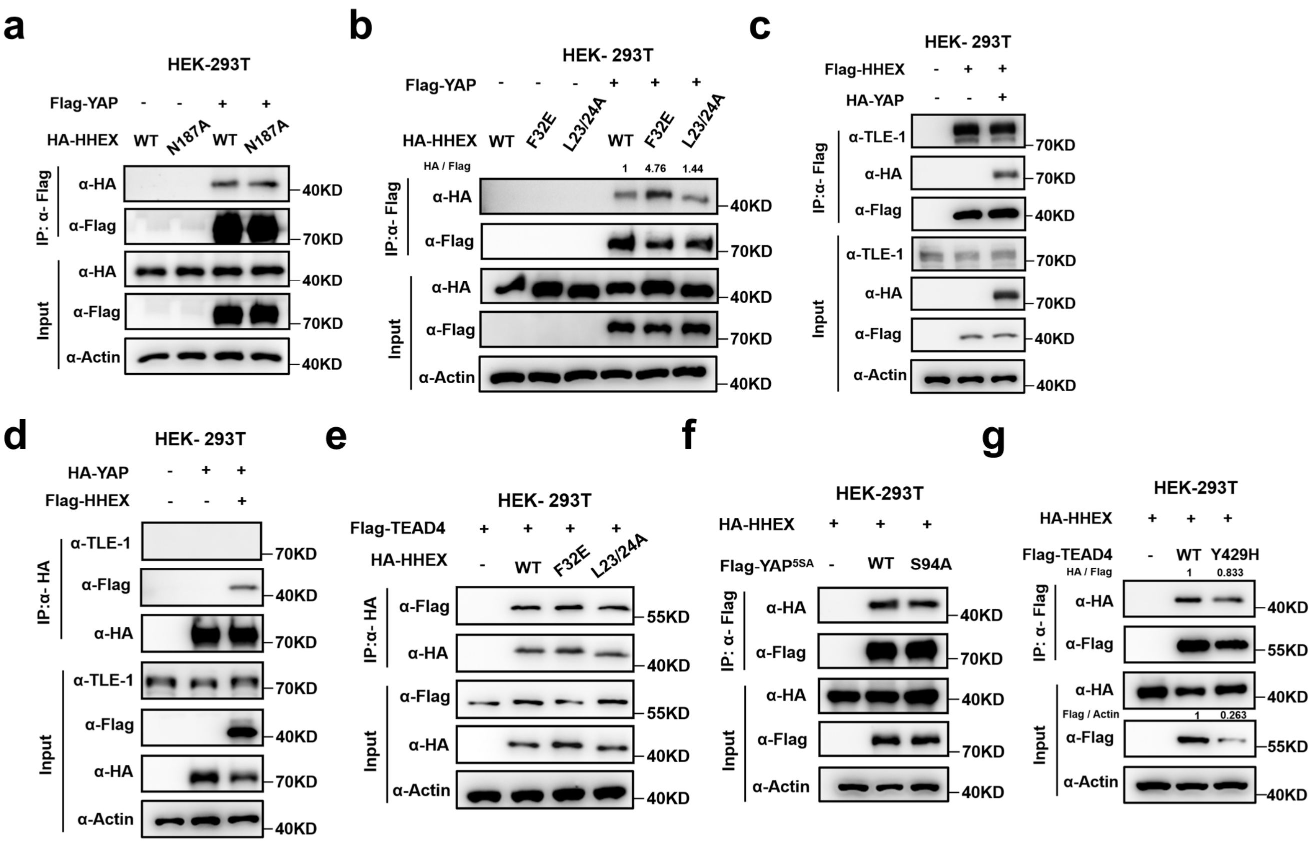

Supplementary Fig. 2. Dissection of the HHEX/YAP/TEAD4 protein complex

a The HHEX N187A mutation did not affect the interaction between HHEX and YAP in HEK-293T cells. **b** The HHEX F32E mutation enhanced but the L23/24A mutant did not affect the interaction between HHEX and YAP in HEK-293T cells. **c** Overexpression of YAP did not promote the interaction between HHEX and TLE-1 in HEK-293T cells. **d** HHEX did not recruit the TLE-1 protein to the YAP/HHEX complex. The interaction between YAP and TLE-1 was not observed in HEK-293T cells transfected with FLAG-HHEX and control plasmids. **e** The HHEX F32E and L23/24A mutations did not affect the interaction between HHEX and TEAD4 in HEK-293T cells. **f** The S94A mutation in YAP did not influence the interaction between HHEX and YAP5SA in HEK-293T cells. **g** The TEAD4 Y429H mutation did not affect the interaction between HHEX and TEAD4 in HEK-293T cells. In these co-IP assays, HEK-293T cells were cotransfected with the indicated plasmids. – indicates no transfection; + indicates transfection. Co-IP was performed with anti-FLAG M2 or anti-HA magnetic beads. Immunoprecipitates were analyzed by western blotting using the indicated antibodies. These data (**a-g**) are representative of 3 independent experiments.

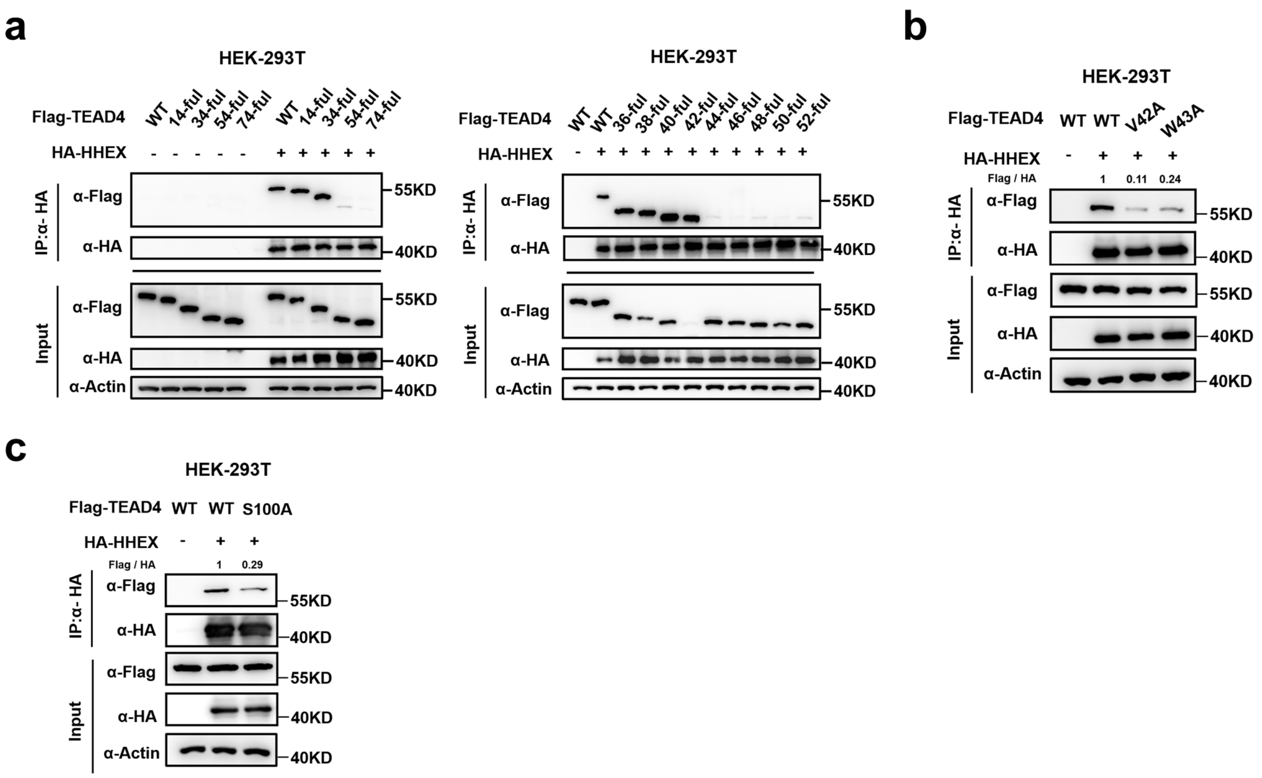

Supplementary Fig. 3 Mapping the interface of TEAD4 which mediates the interaction between TEAD4 and HHEX

a Co-IP of exogenous HA-HHEX and a series of N-terminal truncation mutants of FLAG-TEAD4 in HEK-293T cells. **b** The TEAD4 V42A and W43A mutations significantly attenuated the interaction between HHEX and TEAD4 in HEK-293T cells. **c** The TEAD4-S100A mutant showed a dramatically decreased interaction between HHEX and TEAD4 in HEK-293T cells. In these co-IP assays, HEK-293T cells were cotransfected with the indicated plasmids. – indicates no transfection; + indicates transfection. Co-IP was performed with anti-HA magnetic beads. Immunoprecipitates were analyzed by western blotting using the indicated antibodies. These data (**a-c**) are representative of 3 independent experiments.

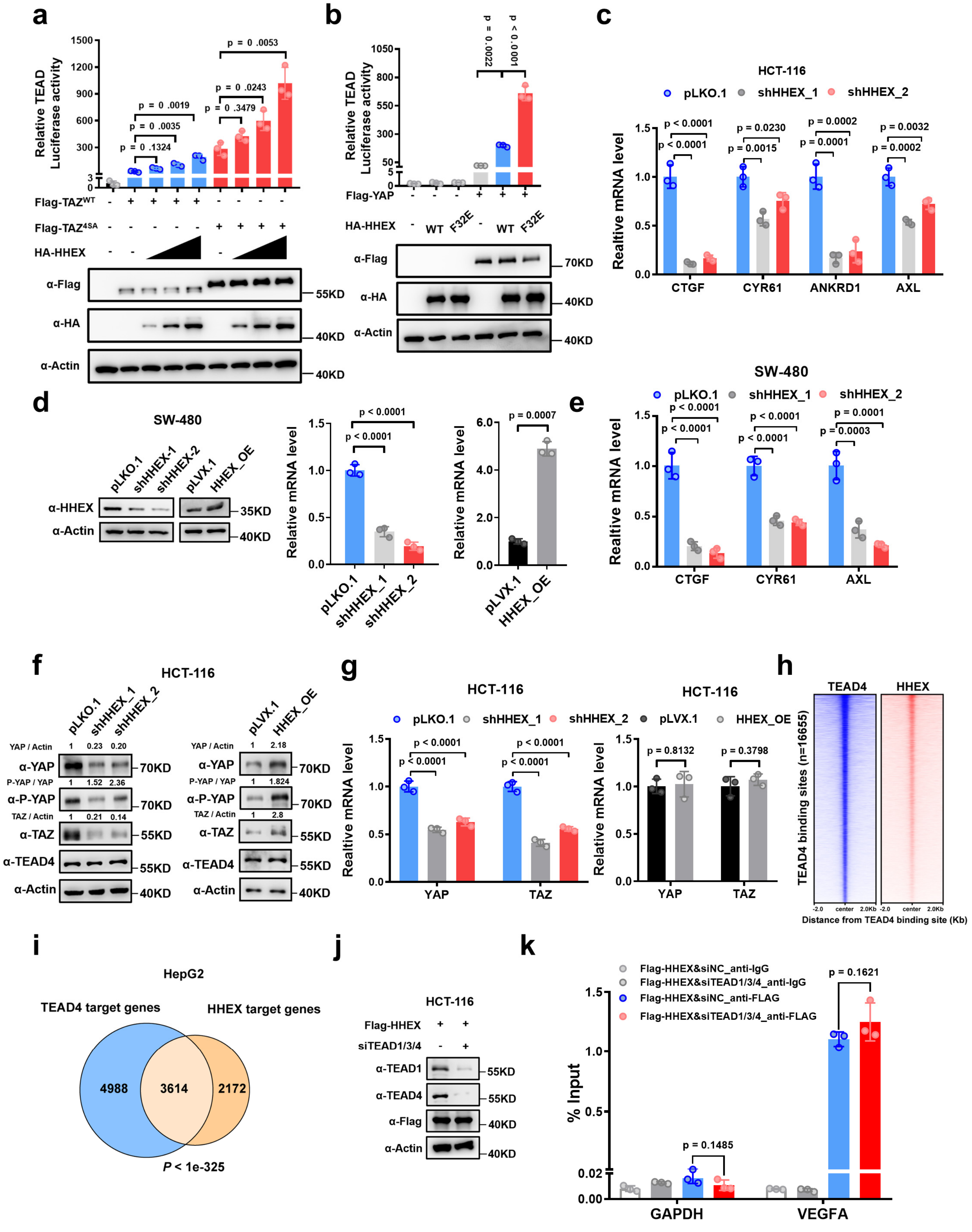

Supplementary Fig. 4. HHEX promotes the transcriptional activity of TEAD

a Overexpression of HHEX enhanced the induction of TEAD luciferase activity by TAZ overexpression. HEK-293T cells were transfected with the indicated FLAG-TAZ, TAZ^{4SA}, and HA-HHEX expression plasmids with a TEAD luciferase reporter plasmid. Firefly luciferase activity was normalized to Renilla luciferase activity. Representative immunoblots of the indicated proteins in total lysates were shown. **b** TEAD luciferase assay of HA-HHEX WT and

F32E in HEK-293T cells. Representative immunoblots of the indicated proteins in total lysates were shown. **c** qPCR analysis of *CTGF, CYR61, ANKRD1* and *AXL* mRNA levels was performed in *HHEX* knockdown HCT-116 cells. **d** Generation of SW-480 cells with *HHEX* knockdown or overexpression. Western blot and qPCR analysis were performed to determine the HHEX protein and mRNA levels. **e** qPCR analysis of *CTGF, CYR61* and *AXL* mRNA levels was performed in HHEX knockdown SW-480 cells. **f** Western blot analysis of YAP, p-YAP, TAZ and TEAD4 in HCT-116 cells with stably HHEX knockdown or overexpression. The data are representative of 3 independent experiments. **g** qPCR analysis of *YAP* and *TAZ* in HCT-116 cells with stably *HHEX* knockdown or overexpression. **h** Heatmap of ChIP-seq data representing TEAD4 and HHEX binding sites in HepG2 liver cancer cells. The heatmap was sorted from the strongest to weakest signal based on TEAD4 binding. **i** Venn diagram showing the overlapping genes with genomic occupancy of both TEAD4 and HHEX in HepG2 liver cancer cells. A hypergeometric test was performed to calculate the statistical significance. **j** Western blot analysis of FLAG-HHEX, TEAD1 and TEAD4 in the nuclear fractions from ChIP assay related with the Figure 2**j**. **k** ChIP-qPCR analysis of FLAG-HHEX binding in gene locus of the reported HHEX target gene *VEGFA* in control and *TEAD1/3/4* knockdown HCT-116 cells. The *GAPDH* locus was used as the negative control. The data are presented as the mean ± SD values. One-way ANOVA with Dunnett's multiple comparison test and Two-tailed Welch's t-test were performed to assess statistical significance for the experiments with >2 groups and 2 groups, respectively, in this figure. n=3 (**a-e**, **g**, **k**) biologically independent samples per group.

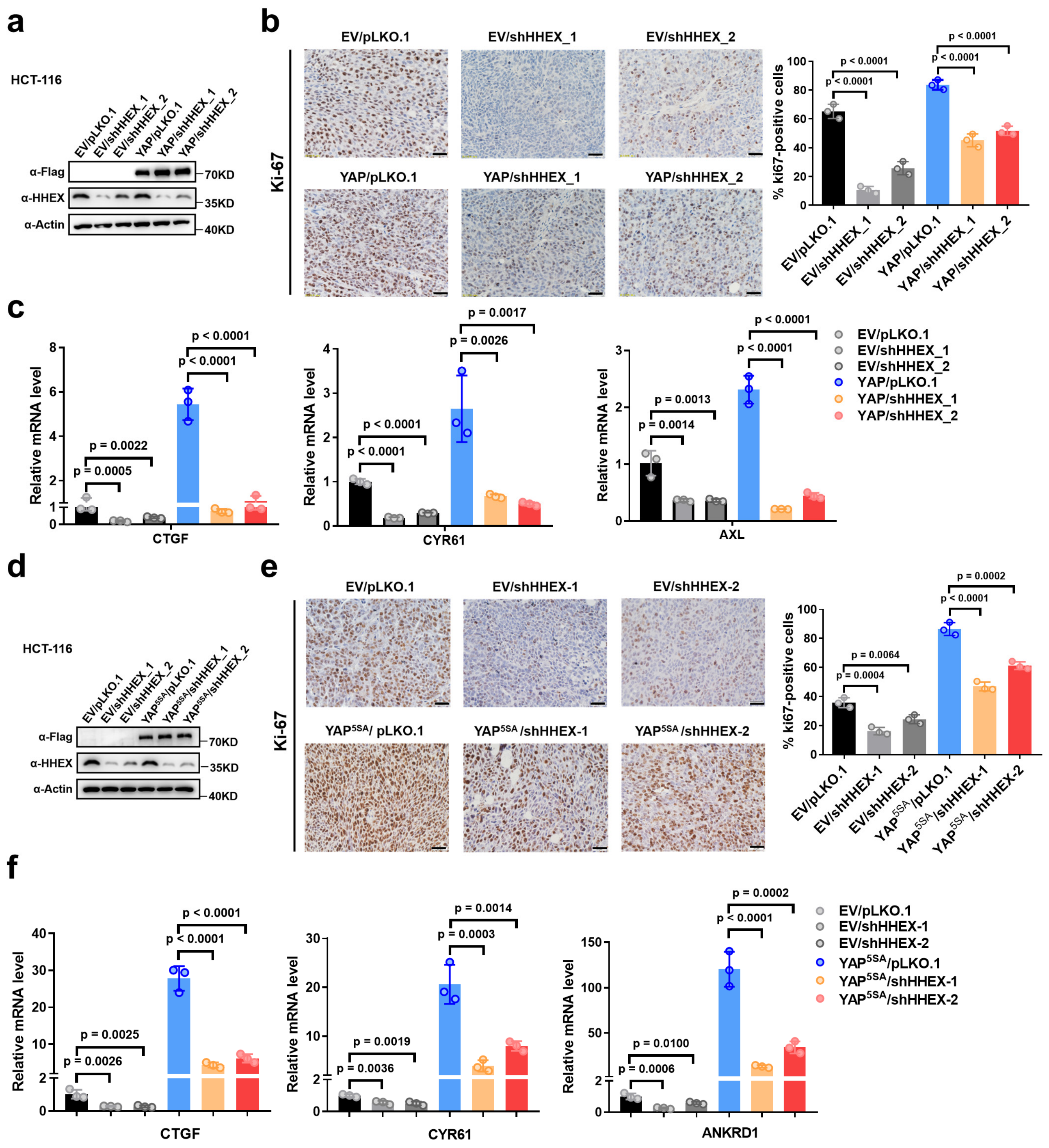

Supplementary Fig. 5. Knockdown of HHEX suppresses the oncogenic function of YAP in HCT116

a Western blot analysis was performed to determine the FLAG and HHEX protein levels in HCT-116 cells stably expressing *HHEX* shRNA and/or YAP WT and in control cells. **b** Representative images of IHC staining of the proliferation marker Ki67 in xenograft tumors derived from HCT-116 cells stably expressing YAP WT and/or *HHEX* shRNA and from control cells. Scale bars, 20 μm. **c** qPCR analysis of *CTGF, CYR61* and *AXL* mRNA levels was performed in HCT-116 cells stably expressing YAP WT and/or *HHEX* shRNA and in control cells. **d** Western blot analysis was performed to determine the FLAG and HHEX protein levels in HCT-116 cells stably expressing YAP5SA and/or *HHEX* shRNA and in control cells. **e** Representative images of IHC staining of the proliferation marker Ki67 in xenograft tumors derived from HCT-116 cells stably expressing YAP5SA and/or *HHEX* shRNA and from control cells. Scale bars, 20 μm. **f** qPCR analysis of *CTGF, CYR61* and *ANKRD1* mRNA levels was performed in HCT-116 cells stably expressing YAP5SA and/or *HHEX* shRNA and in control cells. The data are presented as the mean ± SD values. One-way ANOVA with Dunnett's multiple comparison test was performed to assess statistical significance. n=3 (**b-c**, **e-f**) biologically independent samples per group. These data (**a**, **d**) are representative of 3 independent experiments.

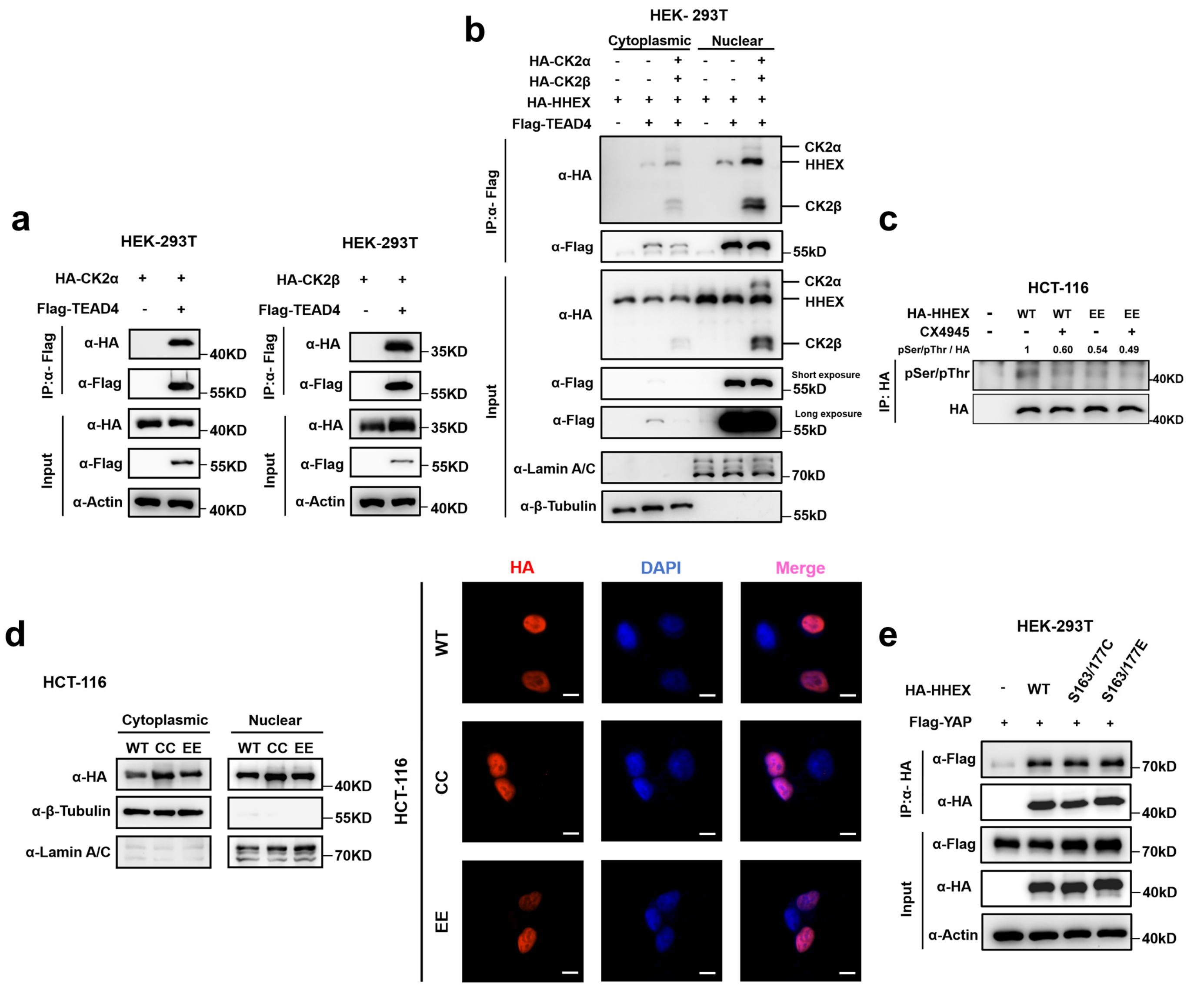

Supplementary Fig. 6. CK2 facilitates TEAD4/HHEX complex formation through phosphorylating HHEX

a Co-IP of exogenous FLAG-TEAD4 and HA-CK2α/β in HEK-293T cells. HEK-293T cells were cotransfected with the indicated plasmids. – indicates no transfection; + indicates transfection. Co-IP was performed with anti-FLAG M2 magnetic beads. Immunoprecipitates were analyzed by western blotting using the indicated antibodies. **b** Overexpression of the CK2α and β subunits significantly increased the interaction between HHEX and TEAD4 in both cytoplasm and nucleus of HEK-293T cells. Cells were fractioned into cytoplasmic and nuclear fractions, then used for co-IP of exogenous FLAG-TEAD4, HA-CK2α/β and HA-HHEX. **c** Western blot analysis of the phosphorylation levels of HHEX WT and S163/177E mutant. HCT-116 cells were transfected with indicated plasmids and treated with the CX-4945 (10 μM) overnight before harvesting for Immunoprecipitation of HA-HHEX. **d** The subcellular localization of HHEX proteins was analyzed by immunofluorescence staining and subcellular fractionation. HCT-116 cells were transfected with plasmids expressing WT, CC mutant or EE mutant HA-HHEX. Cells were fractionated into cytoplasmic and nuclear fractions. Then, the HHEX protein was detected by western blot analysis. Tubulin and Lamin A/C were used as the controls for the cytoplasmic and nuclear fractions, respectively. HCT-116 cells expressing WT, CC mutant or EE mutant HA-HHEX were also analyzed by immunofluorescence staining using an anti-HA antibody (red signal) and DAPI (blue signal). Scale bar, 10 μm. **e** Co-IP of exogenous FLAG-YAP and HA-HHEX WT/CC/EE in HEK-293T cells. These data (**a-e**) are representative of 3 independent experiments.

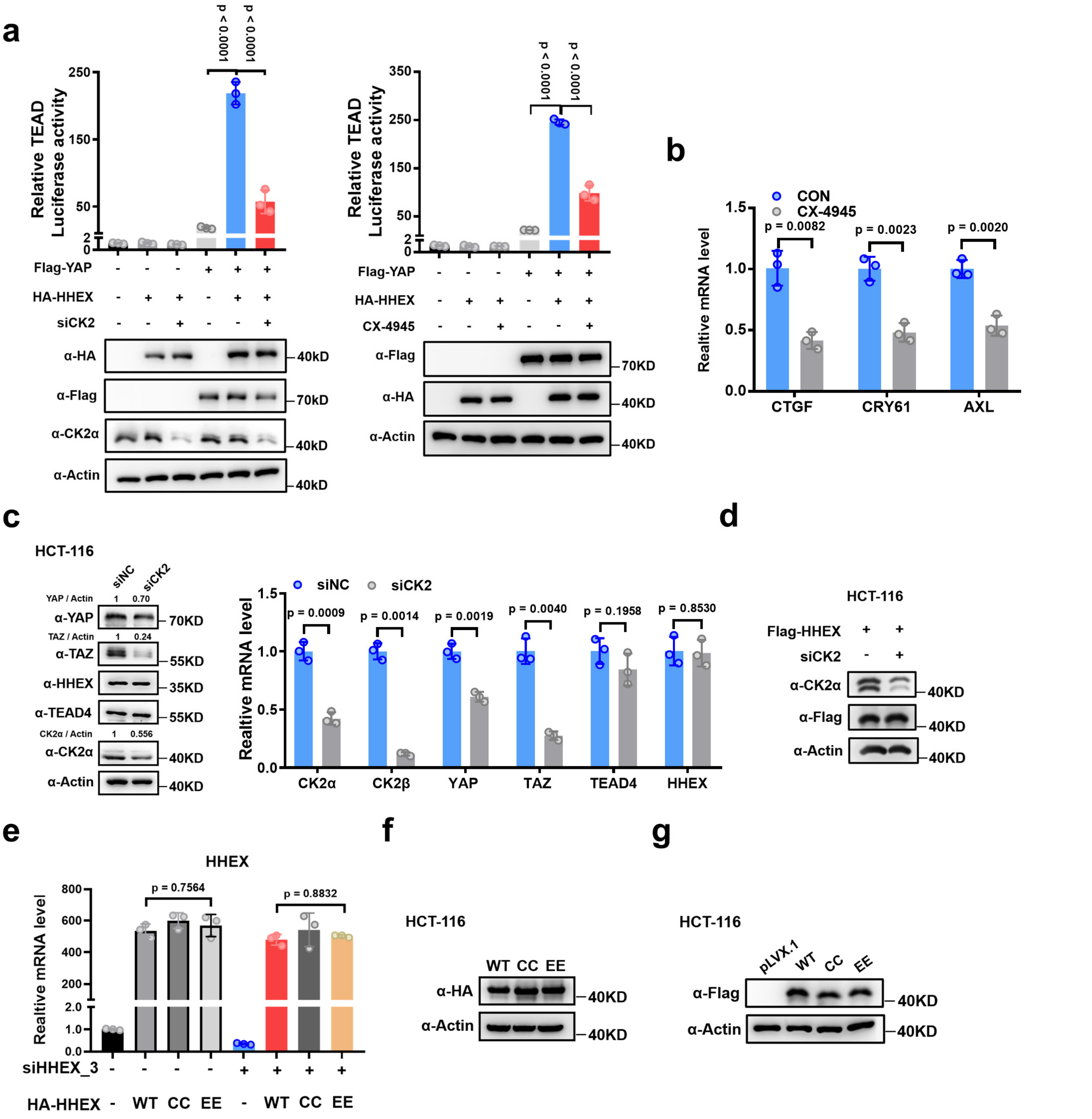

Supplementary Fig. 7. Inhibition of CK2 activity decreases the transcriptional activity of YAP/TEAD

a Knockdown of CK2 and CX-4945 treatment decreased the activation of the TEAD luciferase activity induced by co-expression of YAP and HHEX. HEK-293T cells were transfected with indicated plasmids and siRNA. Luciferase activity was detected after 48 hr. For the CX-4945 treatment, 6 hr after transfection, cells were treated with 10 μM CX-4945 for another 18 hr before detecting the luciferase activity. Representative immunoblots of the indicated proteins in total lysates were shown. **b** qPCR analysis of *CTGF, CYR61* and *AXL* mRNA levels was performed in HCT-116 cells treated with the CK2 inhibitor CX-4945 for 24 hr. **c** Western blot analysis of HHEX, YAP, TAZ and TEAD4 protein levels was performed in CK2 knockdown HCT-116 cells. qPCR analysis of *CK2α, CK2β, HHEX* and *TEAD4* mRNA levels was performed in CK2 knockdown HCT-116 cells. **d** Western blot analysis of HA-HHEX in control and CK2 knockdown HCT-116 cells that were used for ChIP analysis. The nuclear fractions from ChIP process were used for immunoblots. **e** qPCR analysis of *HHEX* in *HHEX* knockdown HCT-116 cells with or without rescued expression of HA-HHEX WT, S163/177C and S163/177E. **f** Western blot analysis of HA-HHEX in HCT-116 cells expressing WT HHEX or the HHEX mutants that were used for ChIP analysis. The nuclear fractions from ChIP process were used for immunoblots. **g** Western blot analysis of FLAG-HHEX in HCT-116 cells stably

expressing WT HHEX or the HHEX mutants that were used for xenograft assay. The data are presented as the mean ± SD values. One-way ANOVA with Dunnett's multiple comparison test (**a, e**) and Two-tailed Welch's t-test (**b, c**) were performed to assess statistical significance. n=3 (**a-c, e**) biologically independent samples per group. These data (**c-d, f-g**) are representative of 3 independent experiments.

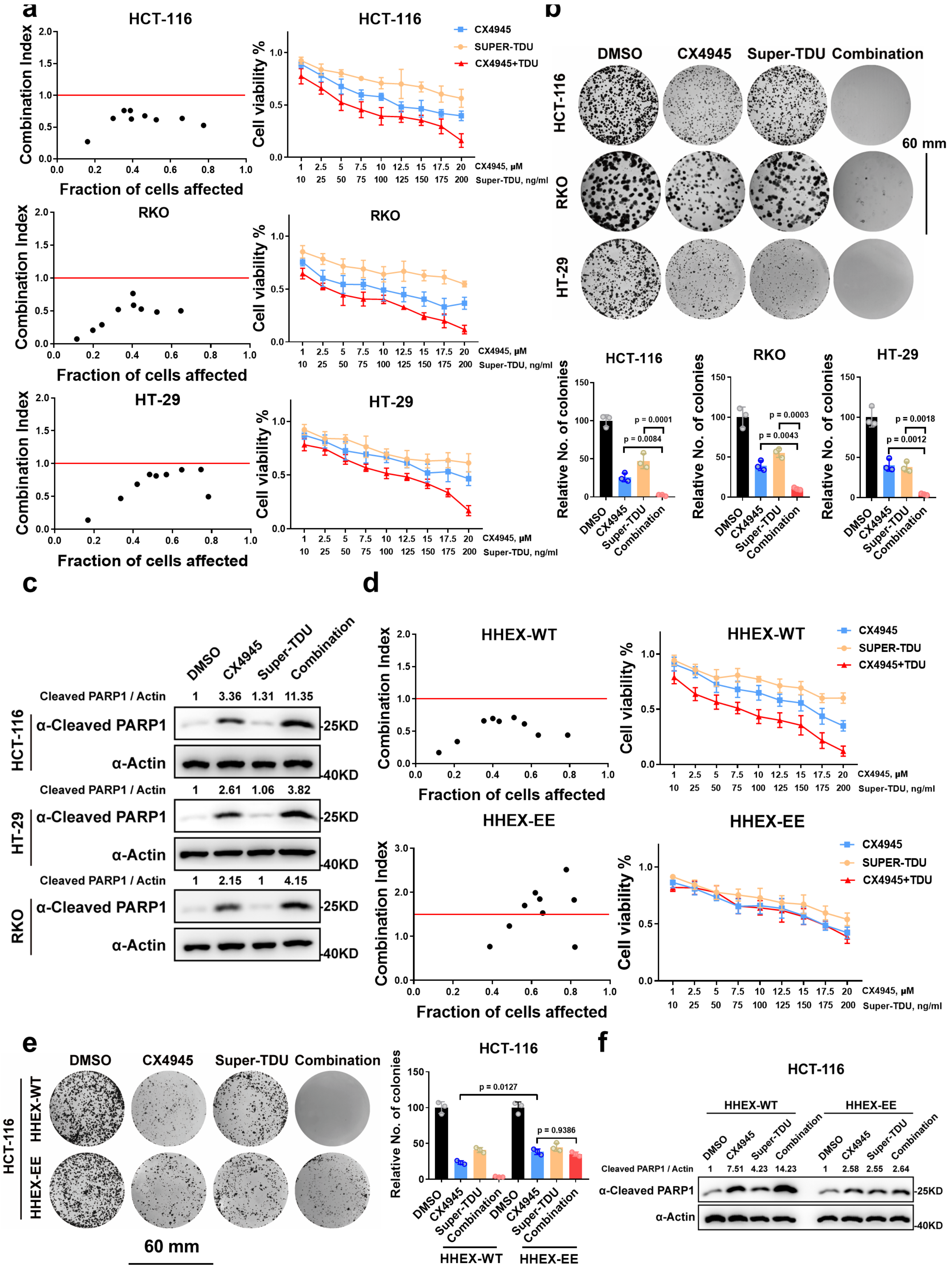

Supplementary Fig. 8. Synergistic antitumor activity of CX-4945 and Super-TDU in CRC

a Cell viability was assessed after 48 h of exposure to the indicated concentrations of CX-4945 and Super-TDU alone or in combination in HCT-116, RKO and HT-29 cells. CCK8 was used to detect the cell viability. CI (combination index) values were calculated using CompuSyn. **b** Representative images of the colony formation

assay. HCT-116, RKO and HT-29 cells were treated with the CK2 inhibitor CX-4945 (10 μM) or the YAP/TEAD inhibitor Super-TDU (100 ng/mL) alone or in combination. Scale bars, 60 mm. **c** Western blot analysis of cleaved PARP1 in HCT-116, RKO and HT-29 cells treated with CX-4945 (10 μM) or Super-TDU (100 ng/mL) alone or in combination for 48 h. **d** Cell viability was assessed after 48 h of exposure to the indicated concentrations of CX-4945 and Super-TDU alone or in combination in HCT-116 stably expressing HHEX WT and S163/177E mutant. CCK8 was used to detect the cell viability. CI (combination index) values were calculated using CompuSyn. **e** Representative images of the colony formation assay. HCT-116 cells stably expressing HHEX WT and S163/177E mutant were treated with the CK2 inhibitor CX-4945 (10 μM) or the YAP/TEAD inhibitor Super-TDU (100 ng/mL) alone or in combination. Scale bars, 60 mm. **f** Western blot analysis of cleaved PARP1 in HCT-116 cells stably expressing HHEX WT and S163/177E mutant. Cells were treated with CX-4945 (10 μM) or Super-TDU (100 ng/mL) alone or in combination for 48 h. The data are presented as the mean ± SD values. One-way ANOVA with Dunnett's multiple comparison test was performed to assess statistical significance. n=3 (**b, e**), n=4 (**a, d**) biologically independent samples per group. These data (**c, f**) are representative of 3 independent experiments.

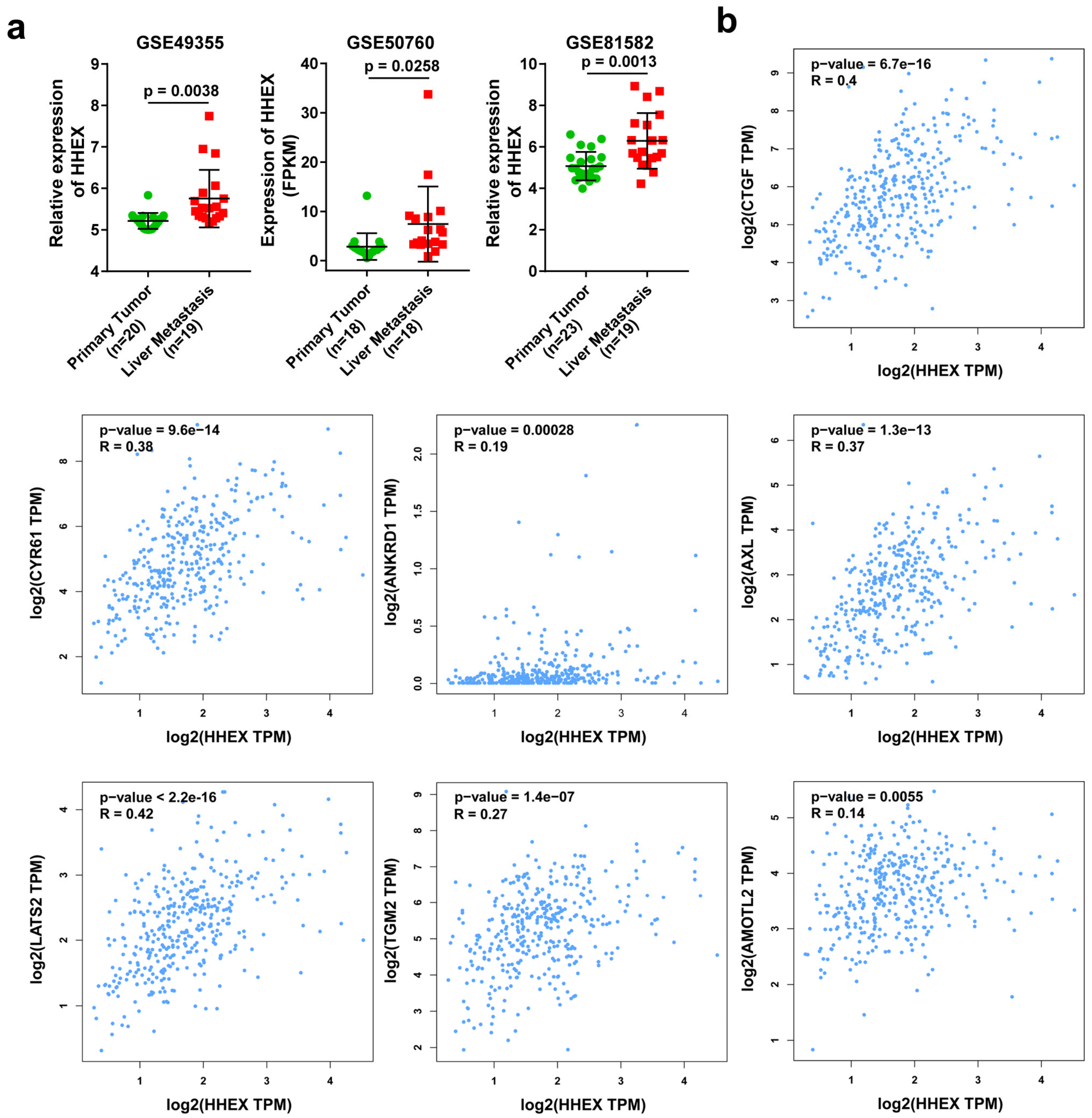

Supplementary Fig. 9. The HHEX expression in metastases and correlation analysis of HHEX expression in CRC

a Differential expression of *HHEX* between primary CRC and metastatic CRC. The mRNA level of *HHEX* was extracted from the public datasets (p=0.0038 for GSE49355, p=0.0258 for GSE50760 and p=0.0013 for GSE81582). n=20 (Primary Tumor), n=19 (Liver Metastasis) of GSE49355. n=18 (Primary Tumor), n=18 (Liver Metastasis) of GSE50760. n=23 (Primary Tumor), n=19 (Liver Metastasis) of GSE81582. The data are presented as the mean ± SD values. Two-tailed Welch's t-test was performed to assess statistical significance. **b** Positive correlations between the mRNA levels of *HHEX* and seven YAP target genes in colorectal cancer (n=367). The mRNA levels of *HHEX* and *CTGF/CYR61/ANKRD1/AXL/LATS2/TGM2/AMOTL2* were extracted from the TCGA-COAD and TCGA-READ datasets. Analysis was performed by the GEPIA2 database (http://gepia2.cancer-pku.cn/#index). Two-sided Pearson correlation analysis was used to assess statistical significance.

Supplementary Table 1. Sequence of shRNA and siRNA used in this study.

shRNA/siRNA	Sequence (5'-3')
shHHEX_1	CCGGTTGGATAGCTCTCAATGTTCGCTCGAGCGAACATTGAGAGCTATCCAATTTTTG
shHHEX_2	CCGGCTGTGATCAGAGGCAAGATTTCTCGAGAAATCTTGCCTCTGATCACAGTTTTTG
siNC	UUCUCCGAACGUGUCACGUTT
siHHEX_1	GUGAUCAGAGGCAAGAUUUTT
siHHEX_2	GGAUAGCUCUCAAUGUUCGTT
siHHEX_3	CCCACUUAAUGGAAAGGCAAA
siCK2α	GAUGACUACCAGCUGGUUC
siCK2β	AAGACUACAUCCAGGACAA
siTEAD1/3/4	UGAUCAACUUCAUCCACAA
siTEAD2	GCCAGAUGCAGUUGAUUCUTT
siYAP_1	GACAUCUUCUGGUCAGAGA
siTAZ_1	ACGUUGACUUAGGAACUUU
siYAP_2	CUGGUCAGAGAUACUUCUU
siTAZ_2	AGGUACUUCCUCAAUCACA

Supplementary Table 2. Sequence of real-time PCR primers used in the study.

Gene	Forward Sequence 5' to 3'	Reverse Sequence 5' to 3'
h-HHEX	AGCTCTCAATGTTCGCCCTC	TCGCCCTCAATGTCCACTTC
h-YAP	ATCCCAGCACAGCAAATTCT	GGATTTTGAGTCCCACCAT
h-TAZ	GGCTGGGAGATGACCTTCAC	CTGAGTGGGGTGGTTCTGCT
h-TEAD4	GTATGAGAGCCCCGAGAACAT	GGATGCGGTAAGAGTAGTGTCC
h-CTGF	CCTGCAGGCTAGAGAAGCAG	TGGAGATTTTGGGAGTACGG
h-CYR61	AAGAAACCCGGATTTGTGAG	GCTGCATTTCTTGCCCTTT
h-ANKRD1	AGTAGAGGAACTGGTCACTGG	TGGGCTAGAAGTGTCTTCAGAT
h-AXL	GTGGGCAACCCAGGGAATATC	GTACTGTCCCGTGTCGGAAAG
h-TGFB2	CCAAAGGGTACAATGCCAAC	CAGATGCTTCTGGATTTATGGTATT
h-CCND1	GCTGCGAAGTGGAAACCATC	CCTCCTTCTGCACACATTTGAA
h-LATS2	ATGAGCTCCACTCTGCTCAATGTCACGG	GCAAGCTTCTCTACCAAGAATGAAAGAGCAT
h-AREG	CGAACCACAAATACCTGGCTA	TCCATTTTTGCCTCCCTTTT
h-CK2α	TGTCCGAGTTGCTTCCCGATACTT	TTGCCAGCATACAACCCAAACTCC
h-CK2β	TTGGACCTGGAGCCTGATGAAGAA	TAGCGGGCGTGGATCAATCCATAA
h-β-ACTIN	GACCTGTACGCCAACACAG	CTCAGGAGGAGCAATGATC
m-Hhex	TCAGAATCGCCGAGCTAAAT	CTGTCCAACGCATCCTTTTT
m-Ctgf	AGAACTGTGTACGGAGCGTG	GTGCACCATCTTTGGCAGTG
m-Cyr61	AGAGGCTTCCTGTCTTTGGC	CCAAGACGTGGTCTGAACGA
m-Ankrd1	GGATGTGCCGAGGTTTCTGAA	GTCCGTTTATACTCATCGCAGAC
m-Gapdh	AGGTCGGTGTGAACGGATTTG	GGGGTCGTTGATGGCAACAA
GAPDH (ChIP)	CGCCCCCGGTTTCTATAAAT	CCTGGCGACGCAAAAGAAG
CTGF (ChIP)	GCCAATGAGCTGAATGGAGT	CAATCCGGTGTGAGTTGATG
CYR61 (ChIP)	AGCAAACAGCTCACTGCCTT	ATGGTAGTTGGAGGGTCGTG
ANKRD1(ChIP)	ATGGCCTGCCACTTTGTTAC	TTTTCAGAACTGGGGTCTGG
VEGFA (ChIP)	AAAGACCCAACTCAAGTATCATCTCCAGG	CACTCACTGTGTGGCCTTAGGTTATTCAAC
