# Supplementary material for: CK2-induced cooperation of HHEX with the YAP-TEAD4 complex promotes colorectal tumorigenesis
Source: Nat Commun. 2022 Aug 25;13:4995. doi: 10.1038/s41467-022-32674-6 (PMC9411202; doi:10.1038/s41467-022-32674-6)
Supplement: Supplementary file 3 — Description of Additional Supplementary Files [file 41467_2022_32674_MOESM3_ESM.pdf]

Description of Additional Supplementary Files

File Name: Supplementary Data 1
Description: Differentially expressed gene of HCT-116 cells with individual knockdown of HHEX, YAP/TAZ and TEAD1/2/3/4.

File Name: Supplementary Data 2
Description: ChIP-seq dataset of TEAD4 and HHEX in HCT-116 and HepG2 cells.
