# Supplementary material for: CK2-induced cooperation of HHEX with the YAP-TEAD4 complex promotes colorectal tumorigenesis
Source: Nat Commun. 2022 Aug 25;13:4995. doi: 10.1038/s41467-022-32674-6 (PMC9411202; doi:10.1038/s41467-022-32674-6)
Supplement: Supplementary file 6 — Reporting Summary [file 41467_2022_32674_MOESM6_ESM.pdf]

nature portfolio

Corresponding author(s): Chen-Ying Liu

Last updated by author(s): Jul 22, 2022

Reporting Summary

Nature Portfolio wishes to improve the reproducibility of the work that we publish. This form provides structure for consistency and transparency in reporting. For further information on Nature Portfolio policies, see our Editorial Policies and the Editorial Policy Checklist.

Statistics

For all statistical analyses, confirm that the following items are present in the figure legend, table legend, main text, or Methods section.

n/a	Confirmed	
☐	☒	The exact sample size (*n*) for each experimental group/condition, given as a discrete number and unit of measurement
☐	☒	A statement on whether measurements were taken from distinct samples or whether the same sample was measured repeatedly
☐	☒	The statistical test(s) used AND whether they are one- or two-sided<br>*Only common tests should be described solely by name; describe more complex techniques in the Methods section.*
☒	☐	A description of all covariates tested
☐	☒	A description of any assumptions or corrections, such as tests of normality and adjustment for multiple comparisons
☐	☒	A full description of the statistical parameters including central tendency (e.g. means) or other basic estimates (e.g. regression coefficient) AND variation (e.g. standard deviation) or associated estimates of uncertainty (e.g. confidence intervals)
☐	☒	For null hypothesis testing, the test statistic (e.g. *F*, *t*, *r*) with confidence intervals, effect sizes, degrees of freedom and *P* value noted<br>*Give P values as exact values whenever suitable.*
☒	☐	For Bayesian analysis, information on the choice of priors and Markov chain Monte Carlo settings
☒	☐	For hierarchical and complex designs, identification of the appropriate level for tests and full reporting of outcomes
☐	☒	Estimates of effect sizes (e.g. Cohen's *d*, Pearson's *r*), indicating how they were calculated

Our web collection on statistics for biologists contains articles on many of the points above.

Software and code

Policy information about availability of computer code

Data collection	No custom computer code or algorithm was used in this study.
Data analysis	The data were anlyzed using Graphpad Prism (v8.0.1), Gelpro 32 (v4.0) , Microsoft Excel 2019 , FastQC (v0.11.9), MACS2 (v2.2.7.1), R package DESeq2 (1.26.0) and ImageJ Launcher (v1.4.3).

For manuscripts utilizing custom algorithms or software that are central to the research but not yet described in published literature, software must be made available to editors and reviewers. We strongly encourage code deposition in a community repository (e.g. GitHub). See the Nature Portfolio guidelines for submitting code & software for further information.

Data

Policy information about availability of data

All manuscripts must include a data availability statement. This statement should provide the following information, where applicable:

- Accession codes, unique identifiers, or web links for publicly available datasets
- A description of any restrictions on data availability
- For clinical datasets or third party data, please ensure that the statement adheres to our policy

Publicly available data used in this paper were obtained from GEPIA colorectal cancer datasets (http://gepia.cancer-pku.cn/index.html). The RNA-seq data and the ChIP-seq data were deposited to NCBI Gene Expression Omnibus (GEO) repository (series GSE196333， https://www.ncbi.nlm.nih.gov/geo/query/acc.cgi?acc=GSE196333).And the human reference genome (GRCh38) was used in ChIP-seq analysis. The ChIP-seq datasets of TEAD4 in HCT-116 (ENCSR000BVJ) and HepG2 (ENCSR000BRP) and HHEX in HepG2 (ENCSR656JZL) were downloaded from ENCODE database. The source data for all the other results are provided as Source Data file with this paper.

Field-specific reporting

Please select the one below that is the best fit for your research. If you are not sure, read the appropriate sections before making your selection.

☒ Life sciences ☐ Behavioural & social sciences ☐ Ecological, evolutionary & environmental sciences

For a reference copy of the document with all sections, see nature.com/documents/nr-reporting-summary-flat.pdf

Life sciences study design

All studies must disclose on these points even when the disclosure is negative.

Sample size

The number of samples for each assay was indicated in each figure legend. For in vitro cellular and biochemical assays, no statistical method was used to determine the sample/group size,however the sample sizes were chosen based on our experience with these assays yielding statistically significant difference between experimental positive and negative controls and on similar sample sizes used in extensive published literature. Results are representatives of at least three biological replicates. For in vivo assays, 5-6 mice were chosen for each condition, this sample size was determined by using power calculation for a t-test difference between two independent means based on a normally distributed population with equal variance.

Data exclusions

No data exclusions in this study.

Replication

A minimum of independent triplicates were carried out for each experiments as described in the legends for each figure.

Randomization

Mice were randomly divided into experimental groups. All other samples collected were used for this study without any discrimination.

Blinding

Blinding was not performed in most of the experiments of this study, as experimental observations would be consistent irrespective of blinding. Investigators who performed animal experiments were not blinded because they needed to prepare the drug or cells freshly. But the Investigators were blinded during the sample collection and data analysis (tumor volume assessment, ex vivo organ imaging and tumor mass collection).

Reporting for specific materials, systems and methods

We require information from authors about some types of materials, experimental systems and methods used in many studies. Here, indicate whether each material, system or method listed is relevant to your study. If you are not sure if a list item applies to your research, read the appropriate section before selecting a response.

Materials & experimental systems

n/a	Involved in the study	
☐	☒	Antibodies
☐	☒	Eukaryotic cell lines
☒	☐	Palaeontology and archaeology
☐	☒	Animals and other organisms
☐	☒	Human research participants
☒	☐	Clinical data
☒	☐	Dual use research of concern

Methods

n/a	Involved in the study	
☐	☒	ChIP-seq
☒	☐	Flow cytometry
☒	☐	MRI-based neuroimaging

Antibodies

Antibodies used

WB antibodies:
HA (Cell Signaling Technology, cat.n.3724; lot 9,dil 1/1000),
Flag (Cell Signaling Technology, cat.n.14793; lot 5, dil 1/1000),
Myc (Cell Signaling Technology, cat.n.2276; lot 3, dil 1/1000),
HHEX (Abcam , cat.n.ab34222; lot GR317796-3, dil 1/1000),
YAP (Santa cruz Biotechnology, cat.n.sc-376830; lot G2721, dil 1/1000),
TAZ (BD Biosciences, cat.n.560235; dil 1/1000),
P-YAP (Cell Signaling Technology, cat.n.13008S; lot 5,dil 1/1000),
TEAD4 (Abcam, cat.n.ab58310; dil 1/1000),
TEAD1 (Abclonal, cat.n.A6768;dil 1/1000),
Pan-TEAD (Cell Signaling Technology, cat.n.13295; lot 2, dil 1/1000),
CTGF (Abclonal, cat.n.A11067; lot 57186, dil 1/1000) ,
CK2α (Proteintech, cat.n.10992-1-AP; lot 00005425, dil 1/1000),
Cleaved parp-1 (Abcam , cat.n.ab32064; lot GR3292031-8, dil 1/1000),
β-actin (Sigma-Aldrich, cat.n.A2228; dil 1/10000),
β-Tubulin (Cell Signaling Technology, cat.n.2128; lot 6, dil 1/1000),
LaminA/C (Cell Signaling Technology, cat.n.4777; lot 5, dil 1/1000),

HRP-labeled Goat Anti-Rabbit IgG(H+L), from Beyotime, cat. no. A0208, dil 1/1000),
HRP-labeled Goat Anti-Mouse IgG(H+L), from Beyotime, cat. no. A0216, dil 1/1000),
IP antibodies:
YAP (Santa cruz Biotechnology, cat.n.sc-376830; lot G2721, dil 1/100),
Pan-TEAD (Cell Signaling Technology, cat.n.13295; lot 2, dil 1/100),
Rabbit mAb IgG Isotype Control (Cell Signaling Technology, cat.n.3900; lot 3),
Mouse mAb IgG Isotype Control (Cell Signaling Technology, cat.n.5415; lot 3).
IHC/IF/PLA antibodies:
HA (Cell Signaling Technology, cat.n.3724; lot 9,dil 1/300 for IF),
Flag (Cell Signaling Technology, cat.n.14793; lot 5, dil 1/300 for IF) ,
HHEX (Abcam , cat.n.ab34222; lot GR317796-3, dil 1/100 for IF and PLA),
YAP (Santa cruz Biotechnology, cat.n.sc-376830; lot G2721, 1/100 for IF and PLA),
TAZ (BD Biosciences, cat.n.560235; dil 1/100 for IF),
TEAD4 (Abcam, cat.n.ab58310; dil 1/100 for PLA),
Cleaved parp-1 (Abcam , cat.n.ab32064; lot GR3292031-8, dil 1/100 for IHC),
Ki67 (Cell Signaling Technology, cat.n.D3B5; lot 6, dil 1/100 for IHC),
Goat anti-Rabbit IgG (H+L) Cross-Adsorbed Secondary Antibody, Alexa Fluor™ 488 (Invitrogen A11008,1:300),
Goat anti-Rabbit IgG (H+L) Cross-Adsorbed Secondary Antibody, Alexa Fluor™ 594 (Invitrogen A11012,1:300),
Goat anti-Mouse IgG (H+L) Cross-Adsorbed Secondary Antibody, Alexa Fluor™ 594 (Invitrogen A11005,1:300).
ChIP antibodies:
HA (Cell Signaling Technology, cat.n.3724; lot 9,dil 1/100),
Flag (Cell Signaling Technology, cat.n.14793; lot 5, dil 1/100) ,
HHEX (Abcam , cat.n.ab34222; lot GR317796-3, dil 1/100),
Normal Rabbit IgG (Cell Signaling Technology, cat.n.2729; lot 3),
Rabbit mAb IgG Isotype Control (Cell Signaling Technology, cat.n.3900; lot 3).

Validation

Most antibodies used are commercially available and have been validated by the respective suppliers. Their validation data are available on the manufacturers websites, as listed below:
WB antibodies verified by supplier:
HA (Cell Signaling Technology, cat.n.3724)
https://www.cellsignal.cn/products/primary-antibodies/ha-tag-c29f4-rabbit-mab/3724?site-search-type=Products&N=4294956287&Ntt=3724&fromPage=plp&_requestid=3269366
Flag (Cell Signaling Technology, cat.n.14793),
https://www.cellsignal.cn/products/primary-antibodies/dykddddk-tag-d6w5b-rabbit-mab-binds-to-same-epitope-as-sigma-s-anti-flag-m2-antibody/14793?site-search-type=Products&N=4294956287&Ntt=14793&fromPage=plp&_requestid=3270909
Myc (Cell Signaling Technology, cat.n.2276),
https://www.cellsignal.cn/products/primary-antibodies/myc-tag-9b11-mouse-mab/2276?site-search-type=Products&N=4294956287&Ntt=2276&fromPage=plp
HHEX (Abcam , cat.n.ab34222; lot GR317796-3),
https://www.abcam.cn/hex-antibody-ab34222.html
YAP (Santa cruz Biotechnology, cat.n.sc-376830),
https://www.scbt.com/p/yap-antibody-g-6?requestFrom=search
TAZ (BD Biosciences, cat.n.560235; dil 1/1000),
https://www.bdbiosciences.com/en-us/products/reagents/microscopy-imaging-reagents/immunofluorescence-reagents/purified-mouse-anti-taz.560235
P-YAP (Cell Signaling Technology, cat.n.13008S),
https://www.cellsignal.cn/products/primary-antibodies/phospho-yap-ser127-d9w2i-rabbit-mab/13008?site-search-type=Products&N=4294956287&Ntt=13008s&fromPage=plp&_requestid=3308726
TEAD4 (Abcam, cat.n.ab58310),
https://www.abcam.cn/tead4-antibody-5h3-ab58310.html?productWallTab=Abreviews&applications=3688&PageSize=10&SortOrder=VoteDesc
TEAD1 (Abclonal, cat.n.A6768),
https://abclonal.com.cn/catalog/A6768
Pan-TEAD (Cell Signaling Technology, cat.n.13295),
https://www.cellsignal.cn/products/primary-antibodies/pan-tead-d3f7l-rabbit-mab/13295?site-search-type=Products&N=4294956287&Ntt=13295&fromPage=plp&_requestid=3311263
CTGF (Abclonal, cat.n.A11067)
https://abclonal.com.cn/catalog/A11067
CK2α (Proteintech, cat.n.10992-1-AP),
https://www.ptgcn.com/products/CSNK2A1-Antibody-10992-1-AP.htm
Cleaved parp-1 (Abcam , cat.n.ab32064),
https://www.abcam.cn/cleaved-parp1-antibody-e51-ab32064.html
β-actin (Sigma-Aldrich, cat.n.A2228),
https://www.sigmaaldrich.cn/CN/zh/product/sigma/a2228
β-Tubulin (Cell Signaling Technology, cat.n.2128),
https://www.cellsignal.cn/products/primary-antibodies/b-tubulin-9f3-rabbit-mab/2128?site-search-type=Products&N=4294956287&Ntt=%CE%B2-tubulin&fromPage=plp
LaminA/C (Cell Signaling Technology, cat.n.4777),
https://www.cellsignal.cn/products/primary-antibodies/lamin-a-c-4c11-mouse-mab/4777?site-search-type=Products&N=4294956287&Ntt=4777&fromPage=plp&_requestid=3313630
IP antibodies verified by supplier:
YAP (Santa cruz Biotechnology, cat.n.sc-376830; lot G2721, dil 1/100),
https://www.scbt.com/p/yap-antibody-g-6?requestFrom=search
Pan-TEAD (Cell Signaling Technology, cat.n.13295; lot 2, dil 1/100),
https://www.cellsignal.cn/products/primary-antibodies/pan-tead-d3f7l-rabbit-mab/13295?site-search-type=Products&N=4294956287&Ntt=13295&fromPage=plp&_requestid=3311263

Rabbit mAb IgG Isotype Control (Cell Signaling Technology, cat.n.3900; lot 3),
https://www.cellsignal.cn/products/primary-antibodies/rabbit-da1e-mab-igg-xp-isotype-control/3900?site-search-type=Products&N=4294956287&Ntt=3900&fromPage=plp&_requestid=6288979
Mouse mAb IgG Isotype Control (Cell Signaling Technology, cat.n.5415; lot 3).
https://www.cellsignal.cn/products/primary-antibodies/mouse-g3a1-mab-igg1-isotype-control/5415?site-search-type=Products&N=4294956287&Ntt=mouse+mab+igg+isotype+control&fromPage=plp
IHC/IF/PLA antibodies verified by supplier:
HA (Cell Signaling Technology, cat.n.3724)
https://www.cellsignal.cn/products/primary-antibodies/ha-tag-c29f4-rabbit-mab/3724?site-search-type=Products&N=4294956287&Ntt=3724&fromPage=plp&_requestid=3269366
Flag (Cell Signaling Technology, cat.n.14793),
https://www.cellsignal.cn/products/primary-antibodies/dykddddk-tag-d6w5b-rabbit-mab-binds-to-same-epitope-as-sigma-s-anti-flag-m2-antibody/14793?site-search-type=Products&N=4294956287&Ntt=14793&fromPage=plp&_requestid=3270909
YAP (Santa cruz Biotechnology, cat.n.sc-376830),
https://www.scbt.com/p/yap-antibody-g-6?requestFrom=search
TAZ (BD Biosciences, cat.n.560235; dil 1/1000),
https://www.bdbiosciences.com/en-us/products/reagents/microscopy-imaging-reagents/immunofluorescence-reagents/purified-mouse-anti-taz.560235
Cleaved parp-1 (Abcam , cat.n.ab32064),
https://www.abcam.cn/cleaved-parp1-antibody-e51-ab32064.html
Ki67 (Cell Signaling Technology, cat.n.D3B5),
https://www.cellsignal.cn/products/primary-antibodies/ki-67-d3b5-rabbit-mab-mouse-preferred-ihc-formulated/12202?site-search-type=Products&N=4294956287&Ntt=d3b5&fromPage=plp
anti-HHEX (Abcam , cat.n.ab34222) antibody used for IHC has been validated in the previous study (K S Wadey et al, 2017).
ChIP antibodies verified by supplier:
HA (Cell Signaling Technology, cat.n.3724)
https://www.cellsignal.cn/products/primary-antibodies/ha-tag-c29f4-rabbit-mab/3724?site-search-type=Products&N=4294956287&Ntt=3724&fromPage=plp&_requestid=3269366
Flag (Cell Signaling Technology, cat.n.14793),
https://www.cellsignal.cn/products/primary-antibodies/dykddddk-tag-d6w5b-rabbit-mab-binds-to-same-epitope-as-sigma-s-anti-flag-m2-antibody/14793?site-search-type=Products&N=4294956287&Ntt=14793&fromPage=plp&_requestid=3270909
HHEX (Abcam , cat.n.ab34222; lot GR317796-3),
https://www.abcam.cn/hex-antibody-ab34222.html
Rabbit mAb IgG Isotype Control (Cell Signaling Technology, cat.n.3900; lot 3),
https://www.cellsignal.cn/products/primary-antibodies/rabbit-da1e-mab-igg-xp-isotype-control/3900?site-search-type=Products&N=4294956287&Ntt=3900&fromPage=plp&_requestid=6288979
Normal Rabbit IgG (Cell Signaling Technology, cat.n.2729; lot 3),
https://www.cellsignal.cn/products/primary-antibodies/normal-rabbit-igg/2729?site-search-type=Products&N=4294956287&Ntt=2729&fromPage=plp&_requestid=6289902

Eukaryotic cell lines

Policy information about cell lines

Cell line source(s)	The HEK-293T,HCT-116,SW-480,HT-29,RKO cell lines were purchased from the American Type Culture Collection (ATCC).
Authentication	Cell lines were authenticated by short tandem repeat (STR) analysis.
Mycoplasma contamination	Cell lines were not tested for mycoplasma contamination but no indication of contamination was observed
Commonly misidentified lines (See ICLAC register)	No commonly misidentified cell lines were used.

Animals and other organisms

Policy information about studies involving animals; ARRIVE guidelines recommended for reporting animal research

Laboratory animals	WT, Hhex flox/+ and Hhex flox/flox male mice were used in this study were in a C57BL/6 background. All the mice used in this study for the xenograft experiments were male BALB/c sathymic nu/nu mice of approximately 4-6 week. Mice were housed in pathogen-free and ventilated cages, and allowed free access to irradiated food and autoclaved water ad libitum in a 12h light/dark cycle, with room temperature at 21±2°C and humidity between 45 and 65%.
Wild animals	No wild animals were used in this study.
Field-collected samples	No field-collected samples were used in this study.
Ethics oversight	All the animal operations were following the laboratory animal guidelines and were approved by the Animal experimentatins Ethics Committee of Xinhua Hospital Affiliated to Shanghai Jiaotong University School of Medicine.

Note that full information on the approval of the study protocol must also be provided in the manuscript.

Human research participants

Policy information about studies involving human research participants

Population characteristics	All clinical sample collection was approved by Xinhua Hospital Ethics Committee, Affiliated with Shanghai Jiaotong University School of Medicine. Informed consent was obtained for the use of all clinical samples. All human CRC and normal tissues were collected in the Department of Colorectal and Anal Surgery, XinHua Hospital, Shanghai Jiao Tong University School of Medicine, from August 2008 to November 2018. A total of 172 paired CRC and normal colorectal tissues were used to prepare tissue arrays. Another 20 CRC samples were collected and sectioned for the WB and qPCR analysis of HHEX expression.
Recruitment	Patients were recruited at admission to the XinHua Hospital, Shanghai Jiao Tong University School of Medicine. Most eligible patients agreed to participation. As a consequence, self-selection bias is low.
Ethics oversight	The study was approved by the ethics committee of the Xinhua Hospital Ethics Committee, Affiliated with Shanghai Jiaotong University School of Medicine.

Note that full information on the approval of the study protocol must also be provided in the manuscript.

ChIP-seq

Data deposition

☒ Confirm that both raw and final processed data have been deposited in a public database such as GEO.

☒ Confirm that you have deposited or provided access to graph files (e.g. BED files) for the called peaks.

Data access links<br>*May remain private before publication.*	https://www.ncbi.nlm.nih.gov/geo/query/acc.cgi?acc=GSE195473
Files in database submission	All files in GEO dataset GSE195473 are the ChIP-seq files for this study.
Genome browser session<br>(e.g. UCSC)	All bigwig files for ChIP-seq data have been deposited to GEO dataset GSE195473 as processed files.

Methodology

Replicates	The ChIP-seq was performed in HCT116 cells.
Sequencing depth	At least 30 million read pairs per samples were sequenced.
Antibodies	HHEX, Abcam, cat.n.ab34222, lot.GR317796-3.
Peak calling parameters	MACS2 (v2.2.7.1) was used to call peaks with the sonicated input as a control and an initial threshold q-value of 0.05 as cutoff.
Data quality	This data quality was assessed using FastQC (v0.11.9).
Software	ChIP-seq reads were aligned to the hg38 reference genome using Bowtie2 (v2.4.1), bigwig files were generated using Deeptools (v3.5.0).
